# Proposed treatment options for combined nevi: report of two cases using combination therapy involving surgical excision and laser therapy

**DOI:** 10.1007/s10103-025-04729-9

**Published:** 2025-11-18

**Authors:** Kojiro Nagai, Yoshihisa Yamagi, Nobuyuki Mitsukawa

**Affiliations:** Department of Plastic, Reconstructive and Aesthetic Surgery, Faculty of Medicine, Chiba University, Chiba, Japan

**Keywords:** Combined nevus, Combination therapy, Surgical excision, Q-switched ruby laser, Laser therapy

## Abstract

Combined nevi are defined as the presence of 2 or more melanocytic cell groups within one lesion. They now refer to all cases of combinations of benign melanocytic nevus, including nevus cell nevi, Spitz nevi, and other melanocytic tumors, as well as blue nevi. In previous reports, treatment options for combined nevus primarily include surgical excision, laser therapy, cryotherapy, and combination therapy. However, there are no established guidelines for selecting the most appropriate treatment. A treatment for a combined nevus should be selected based on the histopathological structure of the combined nevus. On the other hand, laser therapy for combined nevi affects only blue nevus with melanocytosis and nevus spilus. Considering this background, we devise a treatment flowchart for a combined nevus against a blue nevus background. And we adapted this flowchart to two cases of combined nevus and underwent a combination therapy of excision and laser treatment, and neither case appeared to have any complications(e.g., postoperative hyperpigmentation, ectropion) and didn’t recur. The combination therapy of surgical excision and laser treatment is one of the best treatments for combined nevus, considering the cosmetic aspect. We propose a treatment selection flowchart for combined nevus against a blue nevus background.

## Introduction

Combined nevi are defined as the presence of 2 or more melanocytic cell groups within one lesion [1, 2]. And they are uncommon pigmented skin lesions, accounting for about 1–2.5% of melanocytic nevi [3, 4]. Basically, surgical excision might be the first-choice treatment for a combined nevus. However, there is no evidence to support the choice of treatment for them, and we should consider selecting treatment based on which melanocyte cell population constitutes the combined nevus. We have experienced two cases in which relatively good outcomes were achieved with combination therapy. This article describes a new treatment of 2 cases of combined nevus against a blue nevus background and proposes a treatment selection flow for combined nevus.

## Case series

### Case 1

**Fig. 1:**
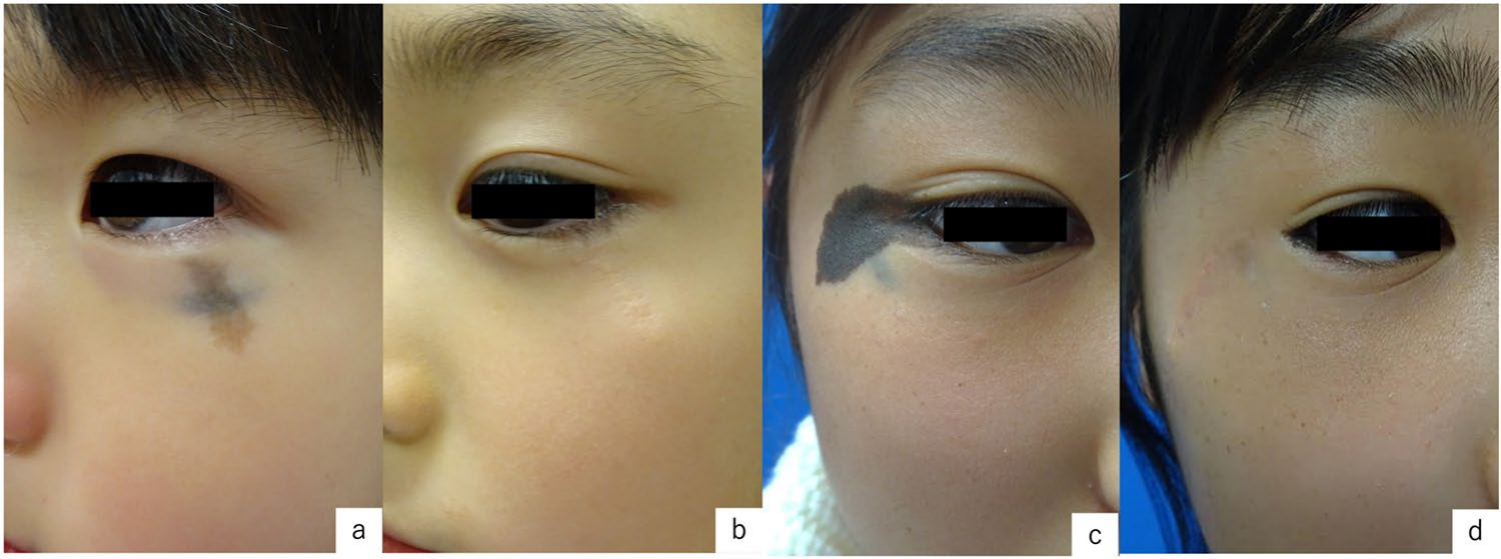
**a**) Case 1 pre-treatment: A 5 × 3 mm horizontal blue spot was observed along the inferior margin of the left orbit, intersected perpendicularly by a relatively homogenous 7 × 4 mm brownish macule extending downward from the lower lash line. **b**) Case 1, 33 months after combination therapy: SEs three times and QSRLs twice **c**) Case 2 pre-treatment: there was a 50 × 20 mm black-brown patch extending outward from the right outer canthus, and a 5 × 3 mm blue patch situated orthogonally at the central lower margin of the lesion. **d**) Case 2, 22 months after combination therapy: SEs twice and QSRLs four times

**Fig. 2:**
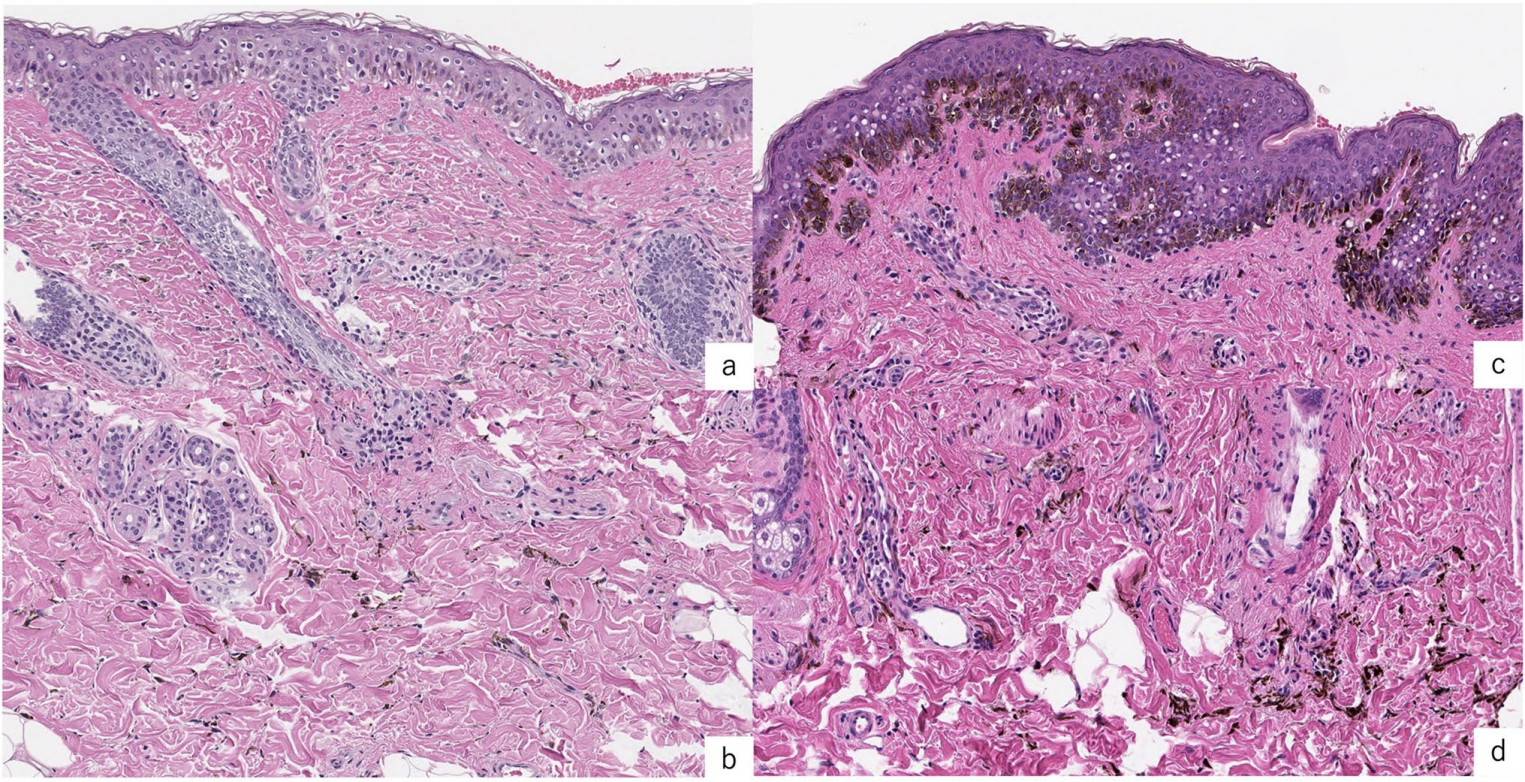
**a**) Case 1, histopathological examination: Nevus cells proliferated locally within the epidermis, exhibiting a partially alveolar pattern. (H&E, original magnification, × 200) **b**) Case 1, histopathological examination: There was increased proliferation of melanocytes, which exhibited spindle-shaped to dendritic morphology in the dermis. (H&E, original magnification, × 200) **c**) Case 2, histopathological examination: There is an increased number of single, monomorphous melanocytes with like circular nuclei above the dermoepidermal junction. Partially, they form an alveolar pattern. (H&E, original magnification, × 200) **d**) Case 2, histopathological examination: Melanocytes containing melanin granules proliferate between collagen fibers in the dermis. (H&E, original magnification, × 200)

A healthy 5-year-old Japanese girl was referred to us for treatment of a lesion on the left lower eyelid, characterized by a bule patch present since birth intersecting with a brownish-brown macule that appeared around the age of one. At the initial examination, a 5 × 3 mm horizontal blue spot was observed along the inferior margin of the left orbit, intersected perpendicularly by a relatively homogenous 7 × 4 mm brownish macule extending downward from the lower lash line.(Fig. 1a) Clinically, she was diagnosed as a combined nevus of blue nevus and nevus cell nevus, and we chose combination therapy of serial excision(SE) and Q-switched ruby laser(QSRL). We obtained informed consent from the patient for the treatment and signed informed consent regarding publishing their data and photographs. She underwent SEs three times and QSRLs twice under general anesthesia, and 30 months later, the combined nevus was completely removed. (Fig. 1b) Histopathological examination showed that nevus cells proliferated locally within the epidermis, exhibiting a partially alveolar pattern. (Fig. 2a) In the dermis, there is a proliferated number of melanocytes which exhibit a spindle-shaped to dendritic morphology. (Fig. 2b) Histopathologic examination also diagnosed a combined nevus, consisting of both a blue nevus and a nevus cell nevus.

### Case 2

An 11-year-old health Japanese girl was referred to us for the treatment of a lesion on her right lower eyelid, which consists of a black spot present since birth combined with a blue patch located in the lower left part. Clinically, there was a 50 × 20 mm black-brown patch extending outward from the right outer canthus, and a 5 × 3 mm blue patch situated orthogonally at the central lower margin of the lesion. (Fig. 1c) She was clinically diagnosed with a combined nevus, consisting of both a blue nevus and a nevus cell nevus. We obtained informed consent from the patient for the treatment and signed informed consent regarding publishing their data and photographs. We underwent combination therapy, including two SEs and four sessions of QSRLs. After 22 months, the combined nevus was almost completely removed. (Fig. 1d) Histopathological examination revealed findings consistent with those in case 1 (Fig. 2cd) and confirmed the diagnosis of a combined nevus, including both blue nevus and nevus cell nevus components.

## Discussion

Combined nevi are relatively rare disease which represent less than 1% of all melanocytic naevi [5]. They are defined pathologically as the presence of 2 or more different melanocytic cell groups within one lesion. Combined nevi can develop at any age, but usually affect younger people, with an average age of about 30 years. They can occur on the trunk and extremities in about 35% each of cases [5]. According to Johanna L. Baran et al., they researched 511 cases of combined nevi, and the most common type of combined nevus is that combined with blue nevus(66% of cases) [6]. When differentiating combined nevi from other melanocytic tumors, the first step is recommended to use non-invasive examination methods such as dermoscopy, skin imaging, and large language models [7, 8], and the next step is recommended to be histopathological examination.

Treatment options for combined nevus include surgical excision, laser therapy, cryotherapy, and combination therapy. According to a past report of combined nevus, there were three cases of combined nevus, of both spilus nevus and blue nevus, which underwent only QSRL every 3 months and had good results from infants and toddlers [9]. According to other previous reports, combined nevi consisting of Spitz nevus and blue nevus, as well as those consisting of Spitz nevus and nevus cell nevus, were treated with surgical excision with no reported instances of relapse [10, 11]. However, no previous reports have focused on the treatment of combined nevi, and there have never been reports of combination therapy involving surgical excision and laser therapy. We have experienced two cases of combined nevus that achieved relatively favorable outcomes with combination therapy using surgical excision and laser treatment. So, we suggest that the combination therapy of SE and laser treatment is one of the best treatments for combined nevus. On the other hand, laser therapy for combined nevi affects only blue nevus with melanocytosis and nevus spilus. A treatment for a combined nevus should be selected based on the histopathological structure of the combined nevus. Therefore, we then propose a treatment flowchart for a combined nevus against a blue nevus background. (Table 1) First, the patient is determined whether it contains a blue nevus with pathologically melanocytosis extending from the upper to lower dermis or not when the patient is clinically diagnosed combined nevus. Next, the diagnosis is a combined nevus consisting of a blue nevus with melanocytosis and nevus spilus, and if laser efficacy is positive, only laser therapy is selected; if laser efficacy is negative, combination therapy is selected. If the diagnosis is a combined nevus consisting of a blue nevus with melanocytosis and another type of nevus, combination therapy is selected. On the other hand, blue nevus without melanocytosis and nevus spilus is diagnosed as a combined nevus, and if laser efficacy is positive, combination therapy is chosen; if laser efficacy is negative, excision is chosen. If the diagnosis is a combined nevus consisting of a blue nevus without melanocytosis and another type of nevus, excision is recommended.

**Table 1:**
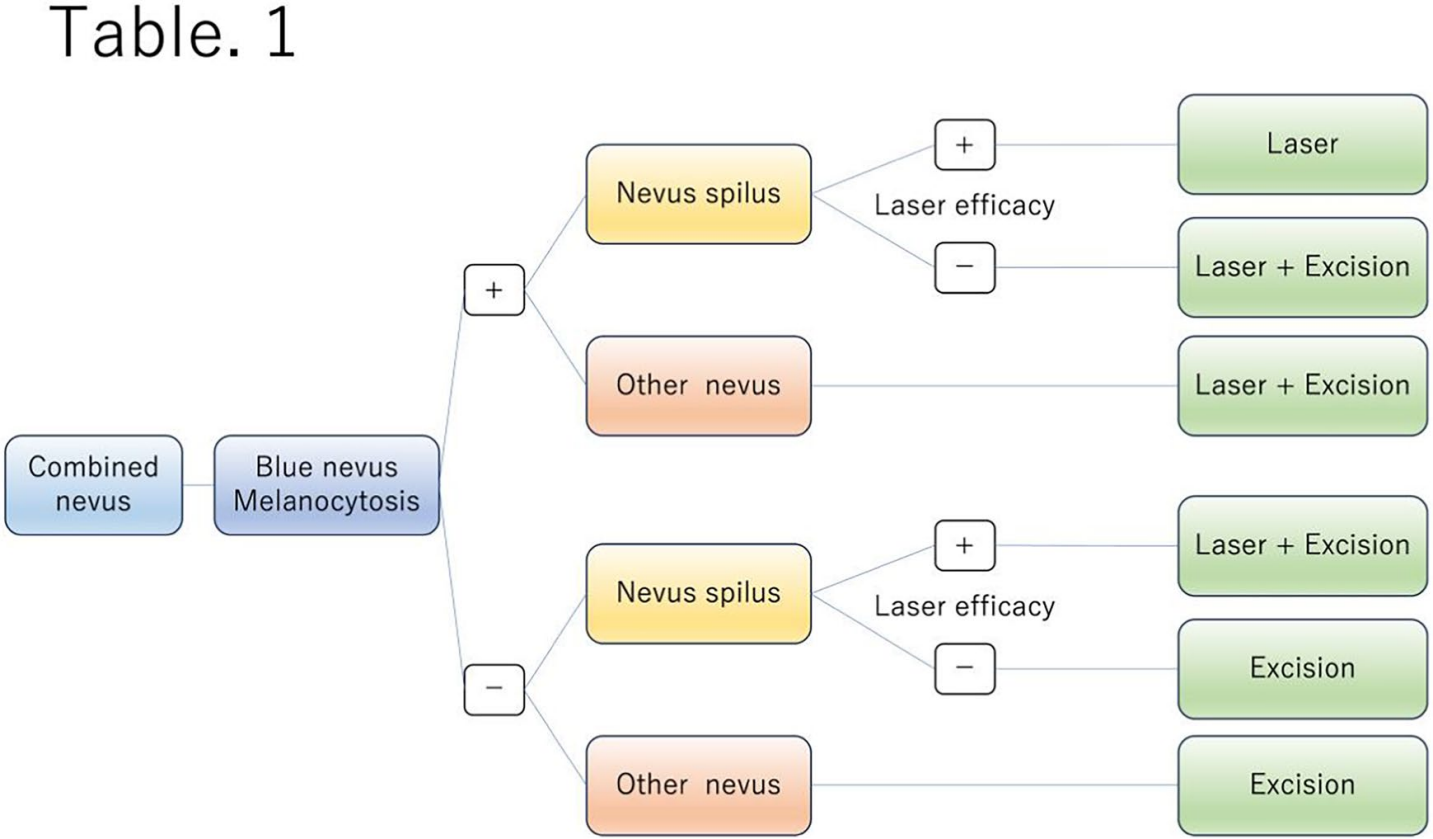
Treatment Flowchart of combined nevus

In our patients, the decision to use combination therapy was primarily based on balancing effective treatment with cosmetic outcomes. Excision alone could cause significant scarring and functional issues such as ectropion, especially in visible areas like the face. Laser therapy alone, while less invasive, is not a complete cure and carries a risk of recurrence. For these reasons, considering the lesion location and the young age of the patients, we chose a combination therapy by this flowchart to achieve both effective treatment and good cosmetic results. As a result, neither case appeared to have any complications(e.g., postoperative hyperpigmentation, ectropion), the scar was not wide or uplifted, and didn’t recur. So, the patient and the patient’s parent were satisfied. If we had only selected SE, the patient’s scars might have been wider or cross-shaped. If we consider using this flowchart, we can aim to treat both the cosmetic aspects and the goal of radical cure.

Lastly, a fundamental understanding of why combined nevi warrant treatment is important. In discussing the significance of treatment, it is essential to recognize that we need to differentiate combined nevi from other melanocytic tumors, that there is diagnostic uncertainty due to overlapping histopathological features, and that, although rare, there is a potential risk of malignant transformation. Additionally, combined nevi can pose particular cosmetic concerns, especially when located on the midface. For these reasons, we believe that treatment has valuable considerations. Accurate diagnosis and appropriate management are crucial, as misdiagnosis could lead to inadequate treatment and unnecessary patient concern.

## Data Availability

No datasets were generated or analysed during the current study.
